# From knowledge to action: Participatory research methods to create an action plan for healthier communities

**DOI:** 10.1371/journal.pone.0347479

**Published:** 2026-05-05

**Authors:** Eva Purkey, Susan A. Bartels, Rifaa Carter, Sophy Chan-Nguyen, Michele Cole, Colleen M. Davison, Meghan Ford, Logan Jackson, Bruce Knox, Autumn Watson, Imaan Bayoumi

**Affiliations:** 1 Department of Family Medicine, Queen’s University, Kingston, Ontario, Canada; 2 Department of Public Health Sciences, Queen’s University, Kingston, Ontario, Canada; 3 Department of Emergency Medicine, Queen’s University, Kingston, Ontario, Canada; 4 Department of Psychology, Queen’s University, Kingston, Ontario, Canada; Caleb University, NIGERIA

## Abstract

**Introduction:**

There is a large knowledge to implementation gap in traditional research. Community based participatory research (CBPR) can fill this gap by improving the validity and relevance of research and by ensuring more effective knowledge translation. CBPR methods can also be used to develop research, policy and programming agendas which can direct the priorities of funders, policy- and decision-makers. The purpose of this paper is to describe the methodology used in a “community engaged” CBPR action planning process, as well as to discuss whether this is an effective method of closing the knowledge to implementation gap. This methods paper illustrates phase two of a CBPR study, the overarching objective of which was to explore how to improve resilience and well being for families facing adversity.

**Methods:**

Using the research findings from a mixed methods multiple case study exploring families’ experiences of adversity and resilience (phase one), as well as extensive community consultation involving over 250 community members, and a CBPR team of 4 academic and 4 community researchers and a research manager, this study employed novel team-based adaptation of reflexive thematic analysis techniques using CBPR principles to develop participatory, evidence-based community action planning.

**Results:**

Over 250 community members participated a total of 11 community meetings as part of the consultation process, producing over 200 action items which the research team then collaboratively organized in 20 final topic areas or themes. Multipronged knowledge translation techniques (infographics, websites, policy briefs) ensured that these findings were communicated back to the community, funders, municipal government and other agencies involved in program planning.

**Conclusion:**

In this context, CBPR was an effective method of engaging a significant number of community members in informing policy, program and research priorities that can improve the lives of people experiencing adversity. Decision makers and program developers demonstrated interest in research findings that involve community participation and consultation by including these findings in subsequent funding applications and referencing them in policy documents. CBPR also led to increased trust and partnership between academic and community organizations as demonstrated by a number of subsequent requests for research training and research partnerships which may benefit all parties in the long run.

## Introduction/Background

### Research to practice gap

There is a well-documented gap between the availability of research knowledge and results, and their implementation into policies, practices and procedures that can improve the health and wellbeing of people and communities [1,2]. Various approaches have been developed to enhance the use of research to inform policy and practice, including integrated knowledge translation [3], implementation science [1,4], and community based participatory research [5–7].

Specifically, community based participatory research (CBPR) has been shown to enhance the validity of research findings, improve knowledge mobilization, and lead to community level actions that enhance community wellbeing [5,8,9]. CBPR engages community members in all aspects of the research process, and as such, increases the likelihood that research will be relevant and useful to communities [10]. Through recognizing community members as experts in their own lives, CBPR can also democratize the creation of knowledge [5,11].

Increasing community participation in setting priorities for research, policy, or health and social services program development can ensure that research findings and/or other types of information are combined with community knowledge to enhance actionable outcomes relevant to community needs [12,13]. If research findings, particularly research in which the community has been involved through the CBPR process, are presented to communities in an accessible manner, and if researchers have built sufficient trust and engagement, then community members can assist in transforming research findings into policy and programming [1,2,12]. Policymakers, people developing and designing health or social service programs, and service providers can use the research-informed priorities generated by a community action plan to ensure research findings can be actioned in a timely manner.

Examples of community participation in priority setting activities include participatory budgeting [14,15], healthcare service priority setting [12,13,16], and participatory development of research agendas [17]. Community action plans are another tool to engage community members and representatives of community organizations in reviewing community data and discussing solutions and interventions to address community-based challenges [18]. Community action plans are a process for identifying and organizing community-driven solutions and directions for community or municipal planning, budgeting, policy and programming. Community action plans can identify which changes are needed, which actors are responsible for implementing change, how change will be implemented, as well as appropriate timelines and resources required [19–22].

We report here on the second phase of a CBPR project designed to identify community priorities for improving the wellbeing and resilience of families with young children who have experienced adversity. The first phase of this study [23], involved a multiple case study analysis of ten families who had experienced adversity in Kingston, Frontenac, Lennox and Addington Counties (KFL&A) in Southeastern Ontario, Canada. Phase One identified themes that either supported or made it more difficult for families with self-described experiences of adversity to thrive. The second phase of this study, reported here, involved knowledge mobilization, or public dissemination and discussion of the results, of the Phase One findings combined with traditional thematic analysis adapted to a CBPR context to inform the development of a community action plan. This paper seeks to answer the question: ‘Can a community engaged CBPR-led participatory approach to action plan development and knowledge mobilization effectively reduce the knowledge-to-implementation gap in research?’. Secondary questions examine the action planning process in itself in terms of effectiveness and output, as well as CBPR as an effective method for community-academic partnership building. It will do this by outlining the methodology used for the community consultation as well as the adapted thematic analysis, and then by exploring initial community level priorities and subsequent activities that will allow the reader to consider whether this methodology was successful in contributing to narrowing this gap within the context of this initiative and possibly into the future.

## Methods

This paper is the product of the second phase of a CBPR Project entitled: Engaging Families to Build Healthy Communities. This project was led by I-CREAte (Innovations for Community Resilience, Equity and Advocacy), a CBPR research team at Queen’s University in Kingston, Ontario. The team includes 4 academic researchers with equity-oriented research portfolios, 4 community researchers, a project manager, a rotating number of students and research assistants, and a community advisory board (CAB) made up of community members and representatives of community-based organizations such as municipal government, community health centers, newcomer organizations, and school boards, among others (see Table 1). This CAB has been an integral part of the I-CREAte team since its inception in 2020 and has provided guidance on all I-CREAte activities. Members have been identified and recruited on an ongoing basis with the objective of representing community members and organizations that can provide representative and insightful input on I-CREAte research activities, and/or that can support dissemination and uptake of research findings. This study was approved by the Queen’s University Health Sciences and Affiliated Teaching Hospitals Research Ethics Board.

**Table 1:** Community advisory board member representation (by sector).

Community members (non-institutional) School boards Immigrant and refugee resettlement programs Universities Municipal government and services Child and youth services Public health services Mental health agencies Healthcare institutions Indigenous community based organizations Social services

This second phase of the research, reported here, involved presenting a subset of Phase One results to various community groups to identify community-generated solutions to the challenges highlighted by participating families in Phase One. Solutions were generated in the context of twelve community meetings, with each community meeting hosted by a member of the I-CREAte CAB.

### Participants

Community meeting participants were made up of either community members, such as service users, or members of various communities within the Kingston, Frontenac, Lennox and Addington region of Southeastern Ontario, or service providers of CAB member organizations. I-CREAte CAB members were invited to host a community meeting and then these meetings were subsequently facilitated by members of the I-CREAte team. The CAB member organizations invited people to attend the meeting from their practice or service community (e.g., service users, community members, or service providers/staff). Meetings were hosted by a number of CAB or CAB-adjacent partners (see Table 2). Approximately 250 participants were recruited between November 1rst 2023 and February 28^th^ 2024 to attend the community meetings which were held between November 2023 and February 2024. All participants were provided with a hard copy and read a verbal consent form or had one interpreted for them on site prior to the meeting taking place by a research assistant. Consent was specifically for the recording of the meetings. No identifying information was collected from any participant, and all participants were explicitly invited to leave prior to the beginning of the meeting or at any time should they not consent to participate or elect not to continue. This process, and this study was approved by Queen’s University Health Sciences and Affiliated Teaching Hospitals research ethics board no. 6034297.

**Table 2:** Community meeting participants and assertions.

Community meeting	Thematic Areas (from Phase One)	Number of participants	Participatory method used
**Local seniors’ association**	Impacts of Covid-19 Social support network Family supports	50 predominantly seniors	World café followed by group discussion
**Community Health Centre**	Healthy Communities Material Deprivation Healthy Systems Navigation	26 community members 5 staff	Facilitated large group discussion
**Coalition of service providers focusing on Adverse Childhood Experiences and resilience (ACEs and resilience coalition)**	Family Supports Healthy Communities Social Support Network	15 service providers	World café followed by group discussion and dotmocracy
**Community based organization providing HIV care, substance use, safe consumption site, low barrier shelter (service users)**	Community Safety Substance Use Material Deprivation	30 community members	Facilitated large group discussion
**Community based organization providing HIV care, substance use, safe consumption site, low barrier shelter (staff)**	Community Safety Substance Use Material Deprivation	10 staff	Facilitated group discussion
**Municipal government**	Material Deprivation Healthy Communities Family Supports	8 staff	Facilitated group discussion
**Local High School**	Impacts of Covid-19 Healthy Communities Social Support Networks	20 high school students	World café followed by group discussion and dotmocracy
**Child and youth community counselling organizations**	Impacts of Covid-19 Social support network Substance use	10 youth and 2 staff	World café followed by group discussion and dotmocracy
**Newcomer refugee resettlement agency**	Anti-Discrimination Integration to Kingston Human Rights	35 government assisted refugee community members broken into 2 linguistic groups	One world café One facilitated group discussion
**Newcomers refugee resettlement agency LGBTQ2 + group**	Anti-Discrimination Integration to Kingston Human Rights	20 newcomers	World café by group discussion
**Urban Indigenous Community**	Healthy Communities Indigenous Community Needs Health and Social system navigation	20 members of urban Indigenous community	Indigenous sharing circle

participants supported by quotes from those participants. A breakdown of community meetings, including host,

participant numbers, and assertions discussed, can be found in Table 2.

### Data collection activities: Community Meetings

Community meetings had flexible but similar structures. Prior to each meeting, the CAB host organization selected three thematic areas (see Table 3 for a list of thematic areas) to be discussed by participants based on the findings from Phase One of the study. Thematic areas were identified by Phase One participants (families) and these were challenges or changes families had identified that helped or hindered the resilience and wellbeing of community members experiencing adversity. Each assertion included a description which was consistent with how the construct had been defined by Phase One participants. Assertion were selected by CAB host member because of their relevance to invited participants. All Phase One assertions were covered by at least one community consultation. Each meeting lasted between 90 and 120 minutes, and began with an overview of the study, including the findings from Phase One. This was followed by an overview of the three assertions selected for discussion with an in-depth review of what the assertion had meant to Phase One

**Table 3:** Phase one thematic areas.

Anti-Discrimination Community Safety Human Rights Substance Use Treatment Healthy Communities Impacts of COVID-19 Indigenous Community Needs Integration into Kingston Material Deprivation Health and Social Services System Navigation Social Support Networks Family Supports

The longest portion of the meeting then involved participant discussion and idea generation. Different tools and methods were used for idea generation depending on the participants involved. Some meetings employed *world cafés*, rotating small group discussions where participants are divided into groups and move from one station to the next. Participants answered questions collectively at each station, building on the reflections of preceding groups [24]. *Dotmocracy*, an interactive voting technique where marks or stickers are used to indicate preference for certain items, was used in combination with this technique in other meetings [25]. Some groups were best organized by facilitated larger group discussions. Following the world café, dotmocracy, or other engagement technique, participants reconvened for an overview and a larger group discussion during which generated ideas were reviewed to ensure that the study team understood what was shared. Ideas were summarized, and the meeting was closed. All non-staff participants were provided with a hot meal and cash honoraria (CAD$50) for their time.

Each meeting generated notes, as well as flip chart paper with descriptions of ideas generated by participants during the meeting. Meetings were audio recorded with participant consent, and recordings were available during analysis for reference. All ideas generated were recorded as notes following the meeting. Ideas were not weighted based on number of times they were suggested or perceived importance, rather all were recorded equally, based on the idea that just because a single person generates a novel idea does not make this idea inherently less important than one that has been more commonly considered.

## Data analysis

### 1. Individual thematic analysis and preparation for group data analysis

Notes from each meeting were compiled separately. One researcher (EP) reviewed each set of community meeting notes, labelled ideas, and synthesized the ideas generated into general themes using inductive thematic analysis [26], which were then shared with the research team. Each idea was then printed on a separate piece of paper, labelled (using color codes) based on the theme, meeting from which it was generated (i.e., participants), and the relevant Phase One assertion to which it was linked.

### 2. Group data analysis

Consistent with the principles of CBPR, the entire research team participated in a group reflexive inductive thematic analysis of all ideas identified by participants [26]. The purpose of this group analysis was to ensure that the community and academic researchers all contributed to the analysis of data generated by the community, recognizing that academic and community researchers bring different expertise based on their different social locations and professional and personal experiences. The group was asked to identify, through data analysis, the priority themes for action identified by community members, and then specific policy or program recommendations within these themes. Braun and Clarke methodology was adapted by the Principle Investigators to allow for joint reflexive analysis by a group of nine academic and community researchers and research assistants (EP, BK, IB, SB, CD, LJ, MC, MF, AW). All members of the group had prior experience with qualitative thematic analysis. All members were involved in all aspects of research design, and had facilitated at least one community meeting. Prior to the group analysis, each member of the group performing the analysis had read the notes from each community meeting. Group analysis took place using the following procedure:

(A) In groups of two, researchers were given all the papers representing ideas pertaining to a certain theme. Each team had several themes to analyze. They were asked to work together to group or re-group these ideas into a second set of broad themes (hereafter topics).
(B) Topics were posted on the wall, with grouped ideas under each. The entire nine-person team worked together to then re-group ideas into thematic groups (hereafter final topic areas). Consensus was achieved through active listening and discussion by members of this research team who had extensive experience working together. This resulted in a three-layered analysis process, involving first one researcher, then two researchers, then the full team of researchers. Twenty final topic areas were identified (see Table 4), each with numerous proposed ideas attached. If an idea applied to more than one final topic area, it was copied and added under each corresponding final topic area.

**Table 4:** Final topic areas and brief description.

**Anti-racism and discrimination**	Within training and education, workplaces, health and social services, and community spaces
**Communication**	At a policy and practice level, fostering collaboration, social support, and accessibility
**Community advocacy**	Intentional community leaders, anti-racism and discrimination, community engagement, municipal accountability
Community building	Urban planning, communication within and between communities, fostering community support
**Cultural safety and trauma informed care**	Embedding trauma informed training and education for services providers and community, ensuring support for youth and for community engagement
**Emergency preparedness**	Fostering systems to enhance community resilience and recovery
**Family supports**	Childcare and healthy child development, community support, family focused community spaces and urban design, family and youth centered programming
**Health care services**	Reconsidering structure of healthcare system, accessibility of services, and expansion of mental health supports all with equity oriented focus
**Human rights**	Increased education initiatives around rights, employment rights, and accessible reporting mechanisms for breaches
**Inclusion**	Multigenerational, multiethnic programming, community services and programs inclusive and specific to all different identities, neighborhood-based diversity programming, specific initiatives for newcomers
**Indigenous**	Priorities around health and social services, and community building and community education initiatives, all with an anti-racism, anti-discrimination and cultural safety and de-colonizing lens
**Legal/justice**	restorative justice, reintegration, community safety
**Material deprivation**	Prioritization of initiatives related to basic needs: housing, food security, accessibility, income, employment, but also mutual aid
**Newcomers**	Broad community inclusion, employment, and language services
**Substance use supports**	Family focused supports, supports for youth, harm reduction approaches, and improved community focused substance use services
**Systems navigation**	Improved logistical support for systems navigation, as well as improved communication about service availability for both community members and service providers
**Training and education**	School supports, parenting supports, life skills and adult learning, technology and media literacy for all
**Transportation**	Enhanced active transportation, improved public transportation, and expansion of transportation between communities particularly for rural residents
**Urban/regional planning**	Access to nature, community gathering spaces, improved transportation, improved safety, and cleanliness of public spaces, and ensuring infrastructure is accessible
**Youth community building**	Accessibility and communication of services/activities/supports, inclusion for youth of diverse backgrounds, enhanced social supports

### 3. Data collation and cleaning

Following the group data analysis, a research assistant transcribed all the final topic areas and ideas into a large spreadsheet. Together, the research team reviewed the spreadsheet line by line. During this process, the following steps were undertaken for data cleaning, analysis and sorting: (1) duplicates were removed and language was standardized as much as possible; (2) each idea was coded to up to three different relevant final topic areas (e.g., anti-racism and anti-discrimination, urban planning, training and education); (3) each idea was also labelled with potential actors who could participate in implementation (e.g., community, community based organizations, municipal government, schoolboard); (4) each idea was labelled with relevant audiences, or groups to which the idea applied (e.g., Indigenous community members, youth, newcomers, general population). It is important to note that while some ideas may have been identified by only one group, all were retained. Just because a single individual or group identifies a possible new idea or intervention does not necessarily make it less important or innovative than ideas that are more common and reported by a larger number of participants. For this reason, the research team explicitly elected to retain all ideas put forward by participants and this paper does not report frequency as an important variable.

## Results

The results of this study fall into two categories. The first involves the concrete evidence-based community informed policy and practice recommendations related to the Community Action Plan. The second type of results relate to the impact of this entire process on the knowledge to implementation gap. These will be addressed separately.

### Action table findings and recommendations

Following data cleaning, the database contained over 200 unique community generated ideas categorized into 20 final topic areas (Table 4). 41 potential actor groups (e.g., municipal government, community-based organizations provincial government, healthcare system), and 36 audiences (e.g., newcomers, youth, Indigenous people) were identified. An example of this work can be found in Table 5. This data was then collated into a searchable database (“Community Action Table”) currently accessible through the I-CREAte project website [27], in which ideas can be sorted based on topic, actor, or audience. Policy briefs and infographics were produced (see example appendix 1), aligning with the topic areas, and shared with CAB members as well as other partner organizations such a funders, through email, social media, in person and virtual meetings [27].

**Table 5:** Example of community action plan data.

Topics (up to three)	Action	Community meeting(s) involved	Potential actors	Audience
**- Anti-racism and discrimination** **- Newcomers**	Promote conversations that raise awareness about the discrimination faced by Black newcomers	-Newcomer refugee resettlement agency	-Community -Community organizations -Newcomer services	General population
**- Healthcare services**	Better trans healthcare with more specialized nursing care, addressing waitlists	-Newcomer refugee resettlement agency	-Health system -Primary care	LGBTQ2 + community
**- Training and Education** **- Youth community building**	Youth diversion in schools	-Local high school	-Schools/school boards -Community organizations	Youth
**- Material deprivation** **- Family supports**	More cooking programs for families (including children in cooking programs)	-Community based organization providing HIV care, substance use, safe consumption site, low barrier shelter (2 meetings: staff and service users) -Phase One participants	-Community -Community organizations -Schools/school boards	Families
**- Indigenous** **- urban/ regional planning**	Ensuring community centre have rooms for smudging (Indigenous cultural practice involving burning of sacred herbs)	ACEs and resilience coalition	Municipal government	Indigenous community
**- Legal/justice** **- Cultural safety and trauma informed approach**	More police in plain clothes with identifying badges during home visits instead of showing up in uniform (which can be triggering)	Urban Indigenous community	Law enforcement	General population
**- Material deprivation** **- Training and education**	Increased access to education on life skills (budgeting, doing taxes, etc)	Phase one participants	Municipal government, provincial and federal government, community organizations	General population
**- Material deprivation** **- Community advocacy** **- Community building**	Supporting mutual aid in neighborhoods	Local high school	Community	General population
**Transportation** **- Youth community building** **- urban regional planning**	Increase public transit access to specific community spaces that are youth oriented	Local high school	Municipal government	Families Youth
**- Cultural safety and trauma informed approaches** **- training and education**	Cultural safety training across all sectors (police, lawyers, children’s aid, healthcare)	Urban Indigenous community, Community health centre, Phase one participants	Community organizations, health system, legal system	Service providers
**- Human rights** **- Antiracism and discrimination**	Increased awareness of employment rights specifically related to racism and discrimination	Newcomer refugee resettlement agency	Community organizations, private sector, unions	General population
**- Indigenous** **- System navigation**	System navigation within Indigenous programs	Urban Indigenous community	Community, community organizations	Indigenous community

### Outputs related to closing the knowledge to implementation gap

In and of themselves, the availability of a Community Action Plan and knowledge dissemination tools does not ensure action or implementation of any recommendations, nor a narrowing of the knowledge to implementation gap. Specific timelines for implementation were not identified by participants, who did not have sufficient policy and funding knowledge to make recommendations. The Action Plan was presented to multiple stakeholder organizations, and these were responsible for identifying actions that were feasible and within organizational scope and using the action table to prioritize initiatives. The Community Advisory Board of the I-CREAte CBPR team has provided some oversight to ensure that the organizations represented on the CAB support implementation and evaluation of the action items, and I-CREAte itself has supported a number of workshops around logic models and program evaluation to support the operationalization of the Action Plan. Although this is more difficult to measure and must be done over a longer term, we would like to outline several of the results related to this work that we believe illustrate the effectiveness of this process in narrowing the research knowledge to implementation gap:

First, since the end of this process, at least one large community organization, a public health unit and a coalition of community organizations have used the Community Action Table findings (website and policy briefs) to support identification of priority activities and/or applications for funding to support future work.

Second, this process has supported increased partnerships between research and community organizations. Specifically, the I-CREAte research team has been invited to deliver bi-annual research workshops for community organizations to support capacity development of community organizations to conduct research and evaluation internally to inform program and policy. Two of these workshops have already taken place and were attended by representatives from eleven community organizations.

Finally, the partnerships developed through this process have led to at least six funding applications designed as collaborations between the research team and community organizations (for example, one already funded related to implementation of Indigenous Systems Navigator in primary care; a second funded project to support integration of refugees into local healthcare systems; and a third a coalition of nine different partner organizations seeking to implement a project related to the prevention of child maltreatment). These funding applications have been directly aligned with priorities identified by community members in the Community Action Plan and, if funded, may lead to meaningful policy and practice change aligned with identified priorities.

## Discussion and knowledge mobilization

### Community planning process

CBPR has a number of important strengths. One is its ability to engage community in the research process. During this two-phased research project, over 250 participants were engaged in identifying challenges and conceptualizing solutions to community issues, including CAB members, families, and a large number of community members. This ability to engage community members in knowledge creation that is useful and relevant to their lives shifts power from academics and institutions to the community, serving as a health intervention in its own right [10,11,28]. In addition, we believe that our application and expansion of CBPR principles is innovative. While community engagement and CBPR are not in their own right, having a large team of community and academic researchers adapt the constructs of reflexive thematic analysis to an interdisciplinary group process is. This builds on previous work of our I-CREAte team in which we adapted Stakes multiple case study methodology for group CBPR analysis. Not only does this allow community researchers to be involved in data analysis and/or feedback, but it allows for the analysis methodology to be actively participatory at each step.

The specific actions recommended by community members may not be extraordinarily novel, however they centre on constructs that are important to families experiencing adversity (the target population in this study), in one specific community. These include basic needs (housing, income, food security), logistical aspects of life (communication, transportation), and equity as a key construct (as illustrated by the diverse target populations identified, the recognition of diverse needs, accessibility as a priority, and need for anti-racism, anti-discrimination, and trauma informed care across all services and initiatives). What is specifically useful in this exercise is the variety of locally relevant, concrete recommendations made by participants, some of which unfortunately seem aspirational at this time and are outside community control (basic income) but some of which can be implemented at local and community levels (changes to municipal transportation, training of service workers). Additionally, many participants expressed support for grass roots, neighborhood led initiatives, and all participants expressed support for community members who were different from themselves, with inclusion and accessibility being key priorities, which is encouraging for future community initiatives.

### Limitations

Whether or not this Community Action Plan leads to meaningful changes in policy and programming remains to be seen. Certainly, there are limitations to this type of engagement. First, depending on the information available to them, community members may or may not be able to prioritize outside of their own lived experience to identify challenges and solutions relevant to the broader community. Second, funders did not attend the community meetings, and with the exception of the municipality, are not CAB participants. As such, solutions were recommended without consideration of funding opportunities and priorities, which often drive (rather than follow) research, policy and programming [29]. This was mitigated in part by meeting with community funders during the knowledge mobilization phase, including the local United Way, and important local actors in public health and health systems, and also by the fact that local organizations who can alter programming and service delivery were active in the project as CAB members, partners in recruitment, and participants themselves. Third, this was not a prioritization exercise. Investigators on the I-CREAte team have experience with community-led priority setting initiatives [17], which have their own limitations. However, in this case, community members were not asked to prioritize interventions, but rather to make comprehensive lists of all their ideas. Our ability to prioritize was partially limited by time, although we also felt that having a wide diversity of perspectives in our final recommendations might increase the likelihood of program and policy-makers being able to identify actions relevant to and feasible for their organizations. This may be open to critique. Fourth, while we endeavored to have a broad cross-section of our community engaged in this research, it did not represent everyone. Specifically, rural voices were absent from this exercise, and better representation of individuals with a variety of intellectual and developmental disabilities could also have been included. While we believe this is an example of robust community engagement, we recognize areas for improvements moving forward. Fifth, we did not have the opportunity to return directly to participants with the knowledge tools produced from this work for further confirmation and clarification. As ideas and recommendations from the Action Plan are taken up for further study or implementation, returning to the community to ensure that their engagement and intent are captured in future planning will be important. Finally, the political and policy environment very often has a significant influence on what communities and organizations choose to prioritize or are able to implement. This changes over time and can make it difficult to persist in implementing a previously agreed upon plan, even when timelines and priorities appeared clear.

### Knowledge to implementation gap

Another strength of CBPR is that it produces research that is relevant, useful, and promptly useable by and in the community. [5–7,30]. Most research methodologies (qualitative, quantitative, etc.) could be used in CBPR with adequate support and in particular time to ensure proper community engagement, capacity building and participation in all phases of the project. Part of the strength of CBPR is determined by the types of knowledge mobilization tools employed, including community meetings, iterative community engagement in the research process, reports, and policy briefs, which are all much more nimble and accessible than traditional peer reviewed publications or presentations at academic conferences. Knowledge mobilization is one of I-CREAte’s top priorities, made possible by long-standing community partnerships. Communities are frequently mined for data without seeing much benefit in return [31,32], which can feel exploitative. It was extremely important to the I-CREAte team the research be used by the community, thereby closing the knowledge to implementation gap. As such, I-CREAte developed a robust knowledge mobilization plan.

First, through the CAB, the entire research process has been co-designed since inception, including both Phase One and Phase Two. As such, the CAB was invested in the knowledge that was being created from the beginning. Second, through the CAB, I-CREAte has direct access to many community-based and other types of organizations and coalitions in the KFL&A region, all of which have extensive networks of their own. The Community Action Plan has been presented to the CAB, who encouraged its use in specific ways, including strategic planning, identification of student or staff research or implementation projects, and communications. Opportunities for communication and knowledge sharing were enhanced by the production of twenty-three plain language infographics (see one in appendix 1), which could be used as social media posts, and as visuals such as posters, waiting room television screens, etc. In addition, targeted policy briefs (appendix 2) were shared with relevant organizations and with particular interest holders (including funders, service providers, and municipal government) who either were not on the CAB but had large networks, or who were on the CAB and requested additional knowledge mobilization meetings. Third, I-CREAte committed to using the findings of this project as the basis of future research, including implementation research, to support community organizations and service providers to implement and pilot ideas identified by the community during the action planning process. Three funded project are already ongoing in this regard, all related to reducing barriers to health system navigation.

### Social participation and partnership

Finally, CBPR is relationship forming. It enhances participation of community members in knowledge creation and, in this case, contributes to the obligation laid out by the World Health Organization for social participation in healthcare [33]. Relationships may have been formed at community meetings, where participants saw each other in new lights. Relationships were formed between CAB members which can lead to additional coordination and partnership between organizations in the future. By creating trust and relationships between academics and the communities they are embedded in and intended to serve, CBPR can increase the opportunities for future participatory implementation research to create new or modify existing programs [9].

The next steps for the I-CREAte team in follow up to this project include identifying future research that is responsive to the priorities identified in the Action Plan. In response to the work completed on this project, I-CREAte obtained community-based funding to support the development of research skills and capacity building within community organizations, and to train additional community researchers, acknowledging the effective academic-community partnerships in this work. I-CREAte is also actively participating in supporting community organizations’ applications for funding for research and programming in line with the Community Action Plan priorities, by providing support for grant writing, implementation of and capacity building around program evaluation frameworks.

## Conclusion

The discussion above illustrates many of the strengths and challenges inherent in a community driven CBPR process. While this study presents an example of a robust CBPR initiative to engage broad cross section of community in research, knowledge mobilization, and planning for their community, there remain multiple limitations to this type of planning. Nevertheless, in this study, many different forms of community engagement were utilized, and traditional research methods, including data analysis, were adapted for CBPR. This initiative has resulted in building relationships between academics, community organizations and the community. These include improved trust, a greater willingness to participate in research initiatives, and a more multi-directional relationship than is often the case (i.e., community reaching in to the academy for support as well as the academy reaching out). Whether the level of participation will be rewarded with implementation of recommendations by program and policymakers remains to be seen, however in our own community, this project has laid the groundwork for future CBPR and implementation studies which have already begun, which we believe will contribute to enhancing community participation, resilience and well-being.
